# Supplementary figures and images for: Effects of a Low-Carbohydrate High-Fat Diet Combined with High-Intensity Interval Training on Body Composition and Maximal Oxygen Uptake: A Systematic Review and Meta-Analysis
Source: Int J Environ Res Public Health. 2021 Oct 13;18(20):10740. doi: 10.3390/ijerph182010740 (PMC8535842; doi:10.3390/ijerph182010740)

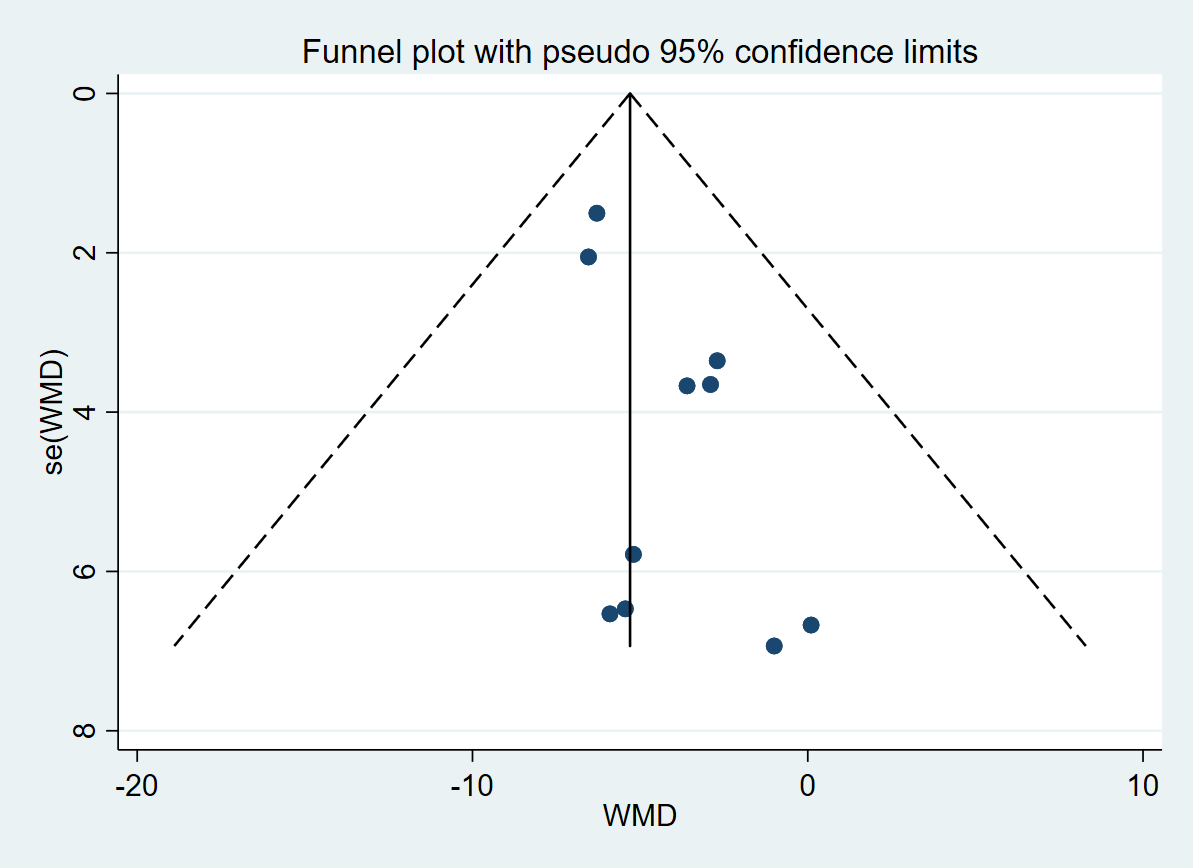

Supplement: Supplementary file 1 [file ijerph-18-10740-s001.zip › ijerph-1375661-supplementary/ijerph-1375661-CN-SI-supplementary/Figures/Supplemental Figure S1.tif]

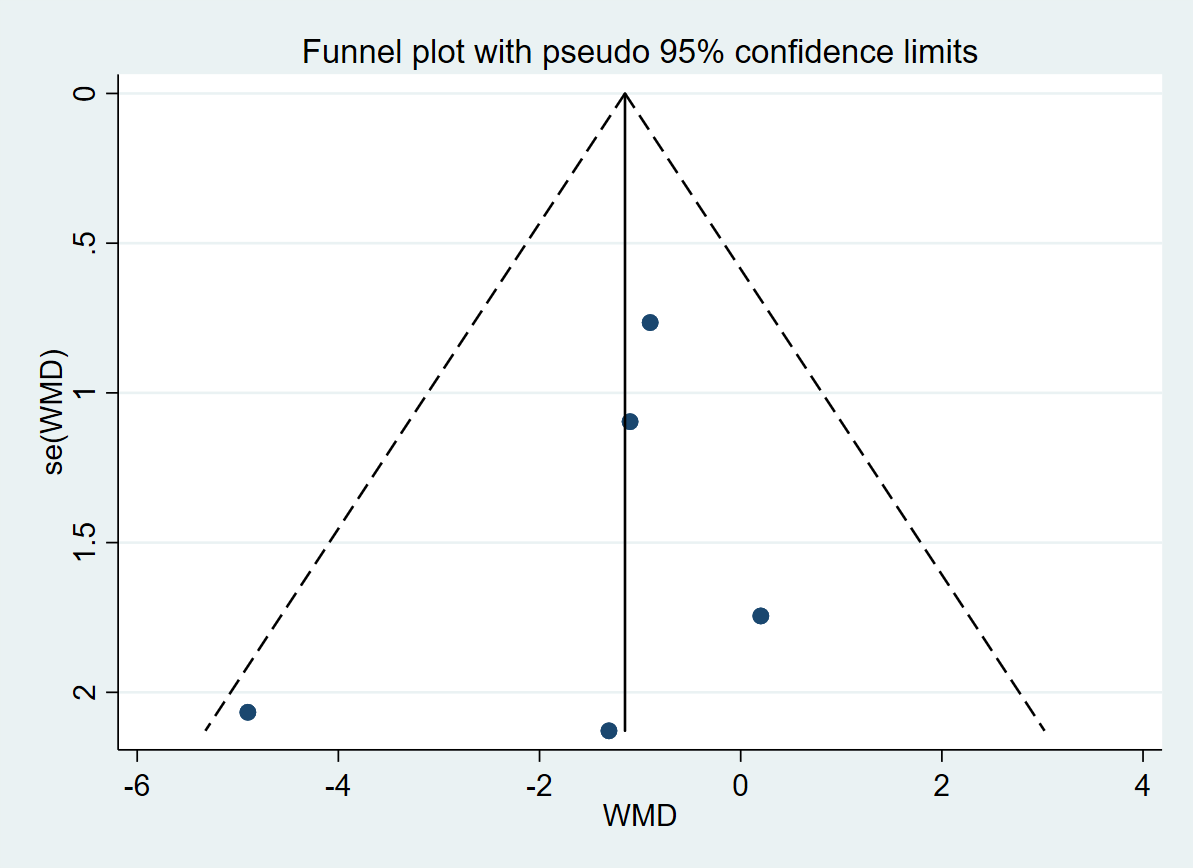

Supplement: Supplementary file 1 [file ijerph-18-10740-s001.zip › ijerph-1375661-supplementary/ijerph-1375661-CN-SI-supplementary/Figures/Supplemental Figure S2.tif]

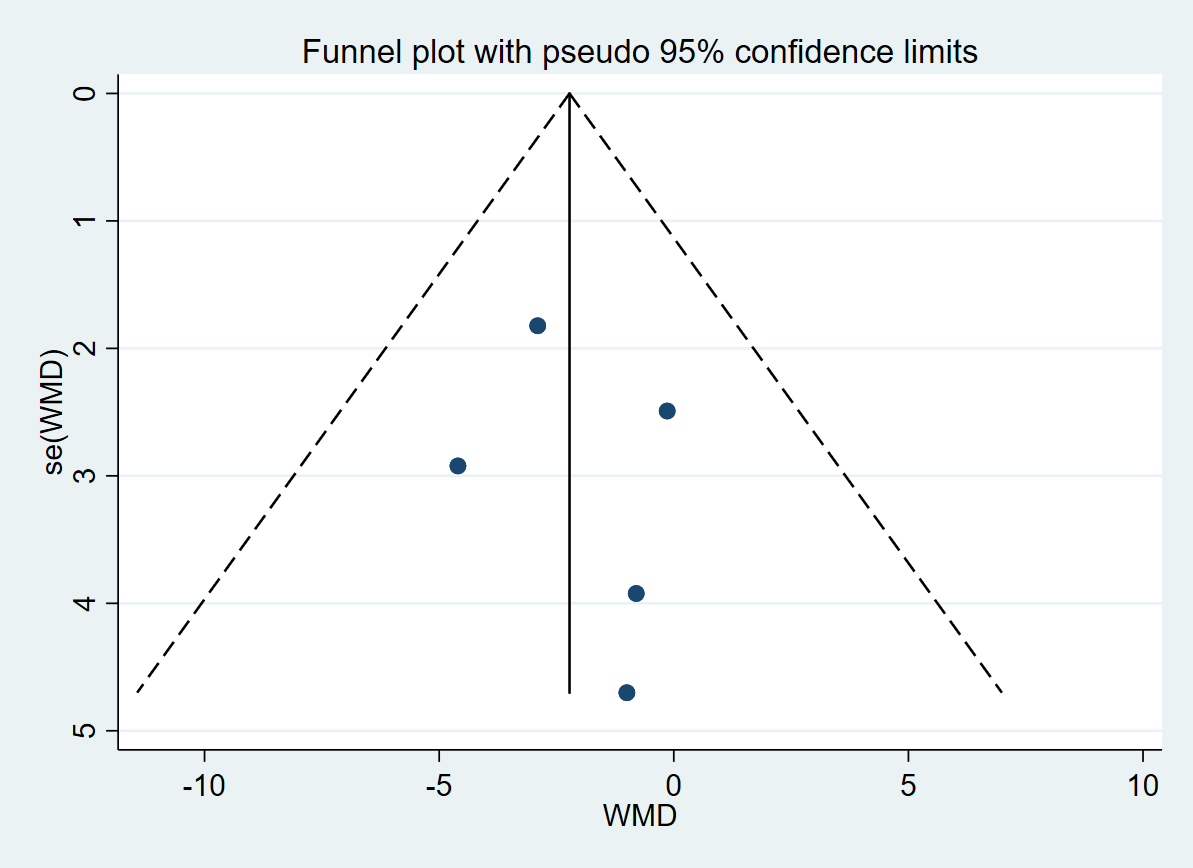

Supplement: Supplementary file 1 [file ijerph-18-10740-s001.zip › ijerph-1375661-supplementary/ijerph-1375661-CN-SI-supplementary/Figures/Supplemental Figure S3.tif]

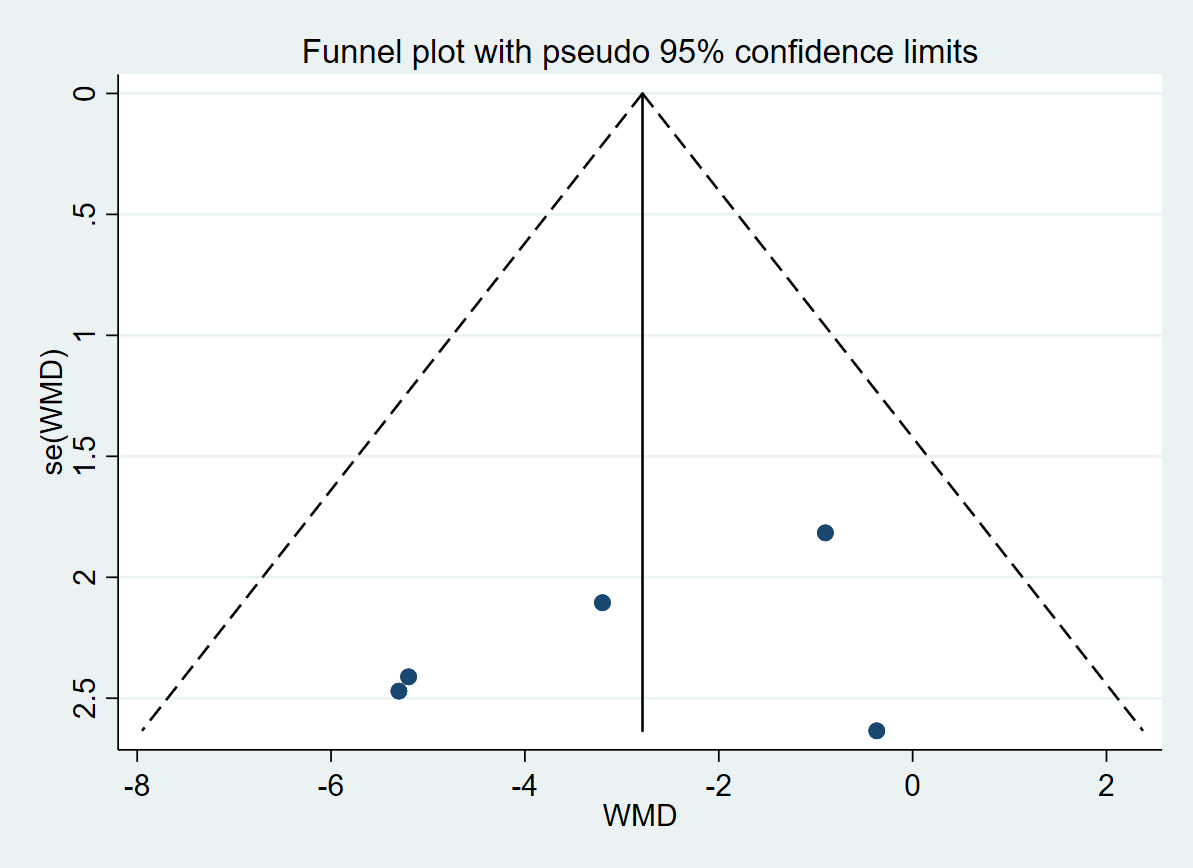

Supplement: Supplementary file 1 [file ijerph-18-10740-s001.zip › ijerph-1375661-supplementary/ijerph-1375661-CN-SI-supplementary/Figures/Supplemental Figure S4.tif]

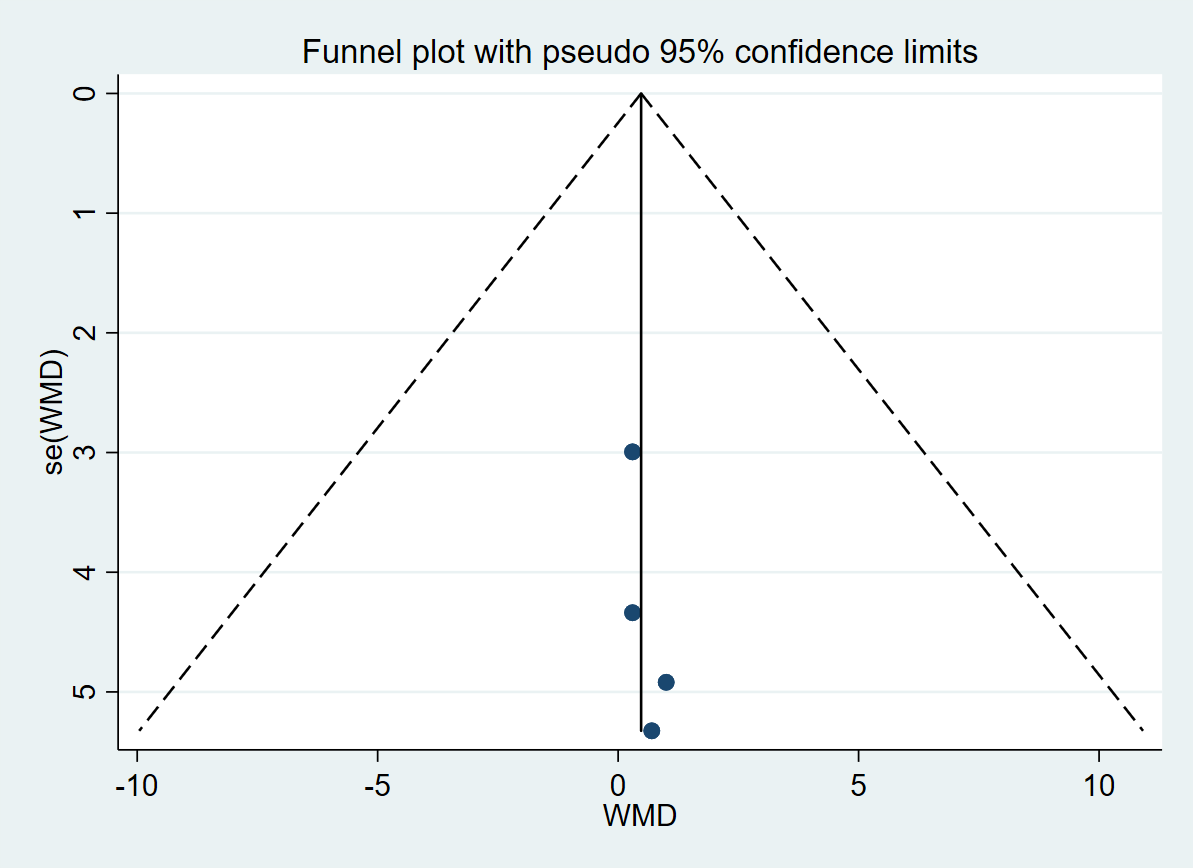

Supplement: Supplementary file 1 [file ijerph-18-10740-s001.zip › ijerph-1375661-supplementary/ijerph-1375661-CN-SI-supplementary/Figures/Supplemental Figure S5.tif]

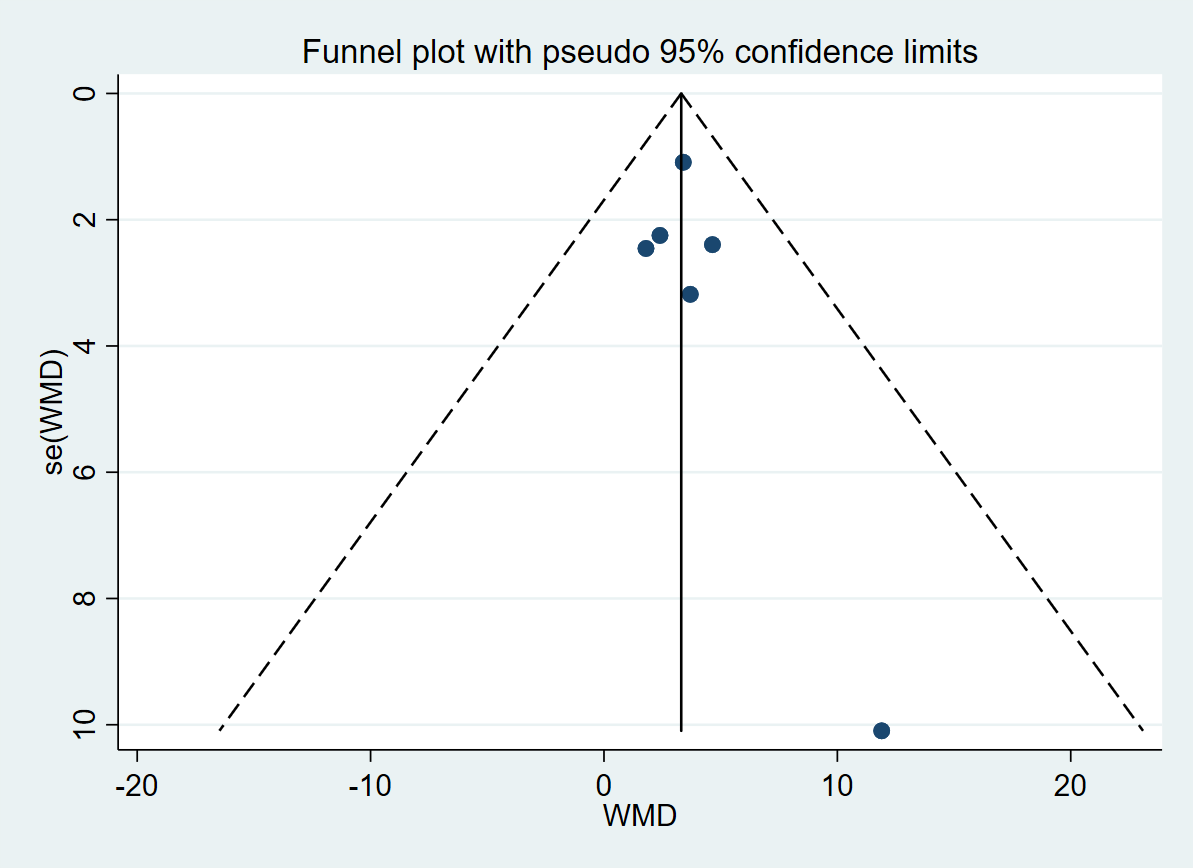

Supplement: Supplementary file 1 [file ijerph-18-10740-s001.zip › ijerph-1375661-supplementary/ijerph-1375661-CN-SI-supplementary/Figures/Supplemental Figure S6.tif]
